# Obesity and blood pressure levels of adolescents in Abeokuta, Nigeria

**DOI:** 10.5830/CVJA-2011-037

**Published:** 2012-06

**Authors:** IO Senbanjo, KA Oshikoya

**Affiliations:** Department of Paediatrics and Child Health, Lagos State University College of Medicine, Ikeja, Lagos, Nigeria; Pharmacology Department, Lagos State University College of Medicine, Ikeja, Lagos, Nigeria, and Academic Division of Child Health, Medical School, University of Nottingham, Derbyshire Children’s Hospital, Derby, UK

**Keywords:** overweight, obesity, central obesity, blood pressure, adolescents, Nigeria

## Abstract

**Background:**

We determined the prevalence of general and central obesity and their relationship with blood pressure levels among adolescents in Abeokuta, Nigeria.

**Methods:**

We selected 423 adolescents from seven schools in Abeokuta, Nigeria, using a multistage random-sampling technique. Body mass index (BMI), waist circumference (WC) and blood pressures were measured.

**Results:**

Twenty-one (5%) children had general obesity and 109 (24.5%) had central obesity. Of those with general obesity, 20 (95.1%) children were centrally obese. With simple linear regression analysis, BMI and WC explained 10.7 and 8.4%, respectively of the variance in systolic blood pressure (SBP), and 3.6 and 2.7%, respectively of the variance in diastolic blood pressure (DBP). Following logistic regression analysis, BMI was the major factor determining SBP levels (OR 0.8, 95% CI: 0.65–0.99, *p* < 0.05).

**Conclusion:**

BMI remains an important anthropometric screening tool for high blood pressure in Nigerian adolescents.

## Abstract

Obesity is a disease in which excess body fat has accumulated to such an extent that the person’s health may be adversely affected.^1^ The International Obesity Task Force (IOTF) has reported that one in 10 children are overweight, with at least 155 million schoolchildren worldwide being affected.^2^ About 30 to 45 million of the overweight children are classified as obese and account for 2–3% of the world’s children aged five to 17 years old.^2^

In the United Kingdom,^3^ Canada^4^ and the USA,^5^ obesity has risen to epidemic levels among children, with the prevalence having more than doubled in the last two to three decades. Under-nutrition is the major nutritional problem in developing countries. Unfortunately, overweight and obesity are now becoming significantly prevalent in developing countries as a result of an environment characterised by easily available, cheap, energy-dense foods, combined with increasingly sedentary lifestyles such as prolonged time spent watching television, playing video games or using computers. This is found particularly in families from a higher socio-economic status in developing countries.^6^,^7^ In India, the prevalence of obesity among adolescent schoolchildren from affluent families was found to be 7.4%.^8^ In Nigeria, among the privileged Nigerian schoolchildren the prevalence of obesity was 18%.^9^

Several epidemiological studies support the relationship between accumulation of body fat and the occurrence of non-communicable diseases such as hypertension and diabetes mellitus.^10^,^11^ More importantly, the accumulation of fat in the central region of the body is a good, proven clinical correlate of increased risk of these chronic diseases.^12^

Traditionally in clinical practice, general obesity is measured using body mass index (BMI). For the estimation of central obesity, there are several anthropometric parameters proposed for its reasonable estimation and they include sub-scapular skin-fold thickness, waist circumference (WC), waist-to-hip circumference ratio, and waist-to-height ratio. Waist circumference measures both the subcutaneous and visceral fat and has been shown to have the most consistent and generally the strongest correlation with adverse lipid concentrations and increased blood pressure levels among children and adolescents.^13^

In developing countries, both children and adult populations are characterised by lean body mass, and a high prevalence of underweight, wasting and stunting. According to Bogin, there is preferential accumulation of fat in the central portion of the body relative to peripheral fat storage in nutritionally stressed populations, with its attendant consequences of hypertension, coronary heart disease and diabetes mellitus.^14^

Information on the pattern of obesity and its influence on blood pressure levels among children in Nigeria is limited. Therefore the aim of this study was to determine the prevalence of general and central obesity and their relationship with blood pressure levels among adolescents in Abeokuta, south-west Nigeria.

## Methods

This was part of a larger study on anthropometric measures and body composition of children and adolescents in Abeokuta, Nigeria.^15^ It was carried out in randomly selected primary and secondary (both public and private) schools in Abeokuta. It was a questionnaire-based, cross-sectional study.

Abeokuta is located on longitude 7° 10″ N and latitude 3° 26″ E and is the capital of Ogun State in south-western Nigeria. It is about 100 km north of Lagos, with an estimated population of four million people. Abeokuta is predominantly a Yoruba city but urbanisation and industrialisation have brought in many other ethnic groups.

Ethical clearance was obtained from the Federal Medical Centre Research/Ethics Committee. Approval of the study came from the Ogun State Ministry of Education.

The teachers, pupils and parents were well informed on the scope and extent of the survey. Consent was obtained from the parents and pupils. At the time of the survey, there was a total of 322 schools in Abeokuta (the ratio of public to private primary schools was 1:1, while the ratio of public to private secondary schools was 3:1). Using the multistage random-sampling technique, seven schools comprising two private and one public primary school, and one private and three public secondary schools were selected by balloting. The basis for this selection was because the number of students in the public primary schools was higher than that in private primary schools, whereas in the secondary schools the numbers were about equal. From each of the selected schools, all grades were studied (primary, grades 1–6; junior and senior secondary, grades 7–12).

On the day of the study, one section of each grade was selected by balloting. Ballot papers were served to all the children in the selected section. These ballot papers were blank except those that were marked with numbers 1 to 15. After all the students had picked a paper, they were asked to open them and those with numbers 1 to 15 were selected. Ninety pupils were selected from each of the seven schools. In all, 630 pupils were selected but only 570 (90.5%) pupils completed the study. The other 60 pupils were excluded based on refusal to participate and evidence of chronic diseases.

Blood pressure was not measured in 147 children aged five to nine years due to non-availability of an appropriate cuff. Each student was interviewed to obtain information on the demographic and socio-economic characteristics of the child’s family. The families were assigned a socio-economic class using the modified method recommended by Oyedeji.^16^

Trained student nurses took all anthropometric measurements. Each measurement was taken by the same examiner to minimise measurement error. The children were weighed using an electronic scale calibrated in 100-g units (SECA/UNICEF, Australia). All children were weighed wearing only underwear and to the nearest 0.1 kg.

The height was measured using a specifically made wooden stadiometer with a steel tape measure. This was done with the child standing erect without shoes and with the eyes looking horizontally and the feet together on a horizontal base. These measurements were done to the nearest 0.1 cm. BMI was calculated by dividing the weight (kg) by the square of the height (m).

Waist circumference was measured midway between the iliac crest and the lowermost margin of the ribs with bare belly and at the end of normal expiration, according to the WHO guidelines.^17^ Standardisation checks on the weighing scale, height boards and tape measure were done periodically during the study period.

For blood pressure measurements, the subjects were seated and rested for five minutes. An appropriately sized cuff, covering at least two-thirds of the upper right arm with the lower border not less than 2.5 cm from the cubital fossa, was applied after restrictive clothing had been removed. Thereafter blood pressure was measured twice using an automatic blood pressure monitor (HEM-712C; Omron, China) and the mean was recorded.

BMI is widely used as an index of general obesity although the cut-off points vary between the 85th and 97th percentiles.^18^ In this study, obesity was defined as BMI at or above the 95th percentile of age- and gender-specific data. A child was regarded as having central obesity if the WC was ≥ the 75th percentile for age- and gender-specific data, as proposed by Fernandez *et al*.^19^ Systolic (SBP) and diastolic blood pressures (DBP) were defined as high when they were ≥ the 90th percentile, according to the Task Force on High Blood Pressure in Children and Adolescents.^20^

## Statistical analysis

Data analysis was by descriptive and inferential statistics, using the SPSS for Windows software version 13. The means and standard deviations (SD) of the weight, height, BMI, WC, and SBP and DBP were calculated according to gender. Gender differences in anthropometric and blood pressure values were compared using the independent-samples *t*-test, while proportions and ratios were compared using the Pearson Chi-squared (χ^2^) test. Simple and multiple logistic regression analyses were carried out on BMI and WC using blood pressure as the dependent variable. A probability (*p*) value of less than 0.05 was accepted as statistically significant.

## Results

A total of 423 subjects with ages ranging from 10 to 19 years had complete data sets, which were analysed. The mean age was 13.2 ± 2.41 years and 233 (55.1%) were males. The social distribution shows that 166 (29.1%), 304 (53.3%) and 100 (17.5%) children belonged to the upper, middle and lower socio-economic classes, respectively. Table 1 shows the means (± SD) of the various anthropometric measures according to gender. All the anthropometric measures increased with age in both genders. The mean weight, BMI and WC were significantly higher in the females (*p* < 0.05, *p* < 0.001, *p* < 0.001, respectively).

**Table 1.** Anthropometric And Blood Pressure Values Of The Study Subjects According To Gender

*Variable*	*Total (n = 423)*	*Male (n = 233)*	*Female (n = 190)*	p*-value*
Age (years)	13. 7 (2.4)	13.7 (2.4)	13.8 (2.4)	0.688
Weight (kg)	39.3 (11.2)	38.0 (11.5)	40.7 (10.7)	0.013
Height (cm)	149.7 (13.6)	150.2 (14.8)	149.1(12.1)	0.418
BMI (kg/m^2^)	17.1 (2.7)	16.4 (2.1)	18.0 (3.1)	0.000
WC (cm)	63.8 (8.5)	61.5 (5.9)	66.7 (10.1)	0.000
SBP (mmHg)	106.9 (11.5)	105.9 (10.9)	108.2 (12.2)	0.05
DBP (mmHg)	60.6 (10.4)	59.0 (10.1)	62.7 (10.4)	0.000
BMI > 2 SD	21 (5.0)	6 (2.6)	15 (7.9)	0.012
WC > 75th percentile	109 (25.8)	36 (15.5)	73 (38.4)	0.000
High SBP	11 (2.6)	4 (1.7)	7 (3.7)	0.206
High DBP	14 (3.3)	7 (3.0)	7 (3.7)	0.697

The BMI of the study population ranged from 11.59 to 34.14 kg/m^2^ with a mean value of 17.1 kg/m^2^. Twenty-one (5%) children were obese, 16 (76.2%) of them females, which was statistically significant (*p* = 0.012). WC ranged from 50.5 to 97 cm and 109 (25.8%) children had a WC above the 75th percentile for the population age and gender. There was a significantly higher prevalence of central obesity among females than males (38.4 vs 15.5%, *p* = 0.000). Twenty (95.2%) of the children who had a BMI above the 90th percentile had a WC above the 75th percentile (χ^2^ = 55.8, *p* < 0.001).

Both SDP and DBP increased significantly with age (*r* = 0.341, *p* = 0.000; *r* = 0.193, *p* = 0.000, respectively). The mean DBP was significantly higher in females (62.7 vs 59.0 mmHg, *p* < 0.001). Eleven children (2.6%) had high SBP and another 14 (3.3%) had high DBP. There was no significant gender difference in the prevalence of high SBP and high DBP (*p* = 0.206, *p* = 0.697, respectively).

The weight, height, BMI and WC had a positive and statistically significant correlation coefficient with SBP and DBP (*r* = 0.126–0.421, *p* < 0.05). The correlation coefficient of BMI with SBP was higher than that of WC with SBP (0.327 vs 0.29). Similarly, the correlation coefficient of BMI with DBP was higher than that of WC with DBP (0.189 vs 0.129).

Table 2 show the relationship between general obesity and blood pressure. There was a significantly higher prevalence of high SBP among male children with general obesity (χ^2^ = 36.5, *p* < 0.001). Among the children with central obesity, a significantly higher prevalence of high SBP (χ^2^ = 22.3, *p* < 0.001) and high DBP (χ^2^ = 4.1, *p* < 0.042) was seen in only the males (Table 3).

**Table 2.** Relationship Between Body Mass Index And Blood Pressure

	*Systolic blood pressure, n (%)*		*Diastolic blood pressure, n (%)*	
BMI percentile	*Normal*	*High*	*Normal*	*High*
Males				
≤ 90th	225 (99.1)	2 (0.9)	220 (96.9)	7 (3.1)
> 90th	4 (66.7)	2 (33.3)*	6 (100)	0 (0.0)
Females				
≤ 90th	168 (96)	7 (4.0)	169 (96.6)	6 (3.4)
> 90th	15 (100)	0 (0.0)	183 (96.3)	7 (3.7)
Total				
≤ 90th	393 (97.8)	9 (2.2)	389 (96.8)	13 (3.2)
> 90th	19 (90.5)	2 (9.5)*	20 (95.2)	1 (4.8)

*p < 0.05

**Table 3.** Relationship Between Waist Circumference And Blood Pressure

	*Systolic blood pressure, n (%)*		*Diastolic blood pressure, n (%)*	
WC percentile	*Normal*	*High*	*Normal*	*High*
Males				
≤ 75th	197 (100)	0 (0.0)	193 (98)	4 (2.0)
> 75th	32 (88.9)	4 (11.1)*	33 (91.7)	3 (8.3)*
Females				
≤ 75th	113 (96.6)	4 (3.4)	113 (96.6)	4 (3.4)
> 75th	70 (95.9)	3 (4.1)	70 (95.9)	3 (4.1)
Total				
≤ 75th	310 (98.7)	4 (1.3)	306 (97.5)	8 (2.5)
> 75th	102 (93.6)	7 (6.4)*	103 (94.5)	6 (5.5)

*p < 0.05

In a simple linear regression analysis, BMI and WC explained 10.7 and 8.4%, respectively of the variance in SBP, and 3.6 and 2.7%, respectively of the variance in DBP. Each increment in BMI increased SBP and DBP by 0.327 and 0.189 mmHg, respectively, while each increment in WC increased SBP and DBP by 0.29 and 0.164 mmHg, respectively. When the effects of BMI and WC on blood pressure were studied in a multiple logistic regression equation model (Table 4), BMI was significantly associated with high SBP (OR 0.8, 95% CI: 0.65–0.99, *p* < 0.05).

**Table 4.** Multiple Regressions Of Body Mass Index And Waist Circumference As Risk Factors For High Blood Pressure*

	*Beta coefficient*	*Standard error*	p*-value*
Systolic blood pressure			
BMI	–0.223	0.109	0.042
WC	0.029	0.047	0.530
Diastolic blood pressure			
BMI	–0.177	0.118	0.136
WC	0.013	0.053	0.316

*Adjusted for age and gender.

## Discussion

Similar to previous studies from Nigeria,^21^-^23^ the prevalence of overweight and obesity from this study (using BMI as the indicator) was low when compared with children in the UK,^3^ Canada^4^ and the USA.^5^ It was also lower than the prevalence recorded in many North African, Middle Eastern and Latin American countries^24^ and in South Africa,^6^ where the prevalence of overweight and obesity has been rapidly increasing. This finding supports the fact that overweight and obesity is still an emerging nutritional problem affecting children in Nigeria. The few cases of overweight and obese children seen in this study were from families of a high socio-economic class, in contrast with what is seen in developed countries.

In our earlier study on the same population,^25^ there was a high prevalence of under-nutrition, which was associated with a high prevalence of moderate to vigorous physical activity among these adolescents. This suggests that there is a negative balance between energy intake and the energy expended in doing exercise. However, even though the mean BMI in this study was as low as 17.1 kg/m^2^, as much as 25.8% of the children had centrally accumulated fat. This is similar to the findings in the Karimojong children of Uganda^26^ and middle-aged Indians,^27^ and is a characteristic feature of populations with chronic malnutrition. This phenomenon, coupled with adaptation to western lifestyles, could explain the rise in prevalence of non-communicable diseases such as hypertension and diabetes mellitus in these populations.

The prevalence of elevated blood pressure in this study is similar to the 3.7% obtained earlier by Bugaje *et al*.^28^ in Zaria, Nigeria and the 4% obtained by Balogun *et al*.^29^ in Ile-Ife, Nigeria. It is lower than the prevalence of 6.69% in India,^30^ 9.5% in Ilorin,^31^ Nigeria, and 12–23% among adolescents in Quebec, Canada.^32^ The upsurge in the prevalence of overweight and obesity, which varies between populations depending on their lifestyle, socio-economic status and other environmental interactions, has been implicated in the differences in prevalence of high blood pressure at a national and international level.

In this study, general obesity was a good predictor of high SBP in males, and WC was a good predictor of high SBP and DBP in males. Surprisingly, despite a higher prevalence of general and central fatness in females, such strong relationships were not seen. This was different from the findings in other studies where strong relationships existed between BMI, WC and BP, irrespective of gender.^13^,^30^,^32^

In keeping with the work of Dobbelsteyn *et al*.^33^ and Yan *et al*.^34^ on Chinese children, BMI was a better indicator of blood pressure levels when compared with WC. Similarly, among Nigerian adults who were urban civil servants, central obesity as assessed by waist:hip ratio made little contribution to elevated blood pressure levels.^35^ However, in Valencia, Spain,^36^ the waist-to-hip ratio of children significantly contributed to SBP levels, while the relationship between waist-to-hip ratio and DBP was of borderline significance when compared with weight, height and ponderal index. The BMI is a measure of total adiposity. It has the limitation of not been able to distinguish between muscle mass and fat mass and therefore it is difficult to determine which is the most significant contributor to blood pressure variability.

According to Stallones *et al*.,^37^ weight was more important than fatness in predicting systolic blood pressure. However, the mechanism of the relationship between weight and blood pressure is not fully known. In a lean black population, there was a threshold above which weight was related to blood pressure.^38^ This was also observed in a study on urban schoolchildren in India where the prevalence of high SBP suddenly increased beyond a BMI value of 20 kg/m^2^ in boys and 21.5 kg/m^2^ in girls.^39^ This aspect of the assessment was not carried out in our own study.

The limitations of this study include use of the BMI-for-age percentile of the study population to define overweight and obesity rather than the internationally recommended standards. These international standards are designed for developed countries where under-nutrition is not a problem. There is a need to develop a national BMI classification for paediatric populations in Nigeria and other African countries.

## Conclusion

Overweight and obesity is an emerging health problem among adolescents in Abeokuta, Nigeria. However, periodic studies coupled with preventive strategies may be necessary to prevent childhood overweight and obesity in Abeokuta from rising at the alarming rate that is presently experienced globally. Although WC was a more direct measurement of body fat, in this study, BMI was a better predictor of blood pressure levels. Future studies are necessary to validate each of these anthropometric measures against adverse serum lipid profiles and other cardiovascular and metabolic risk factors.
